# Realization of Deep UV Plasmonic Enhancement to Photo Response through Al Mesh

**DOI:** 10.3390/ma13153252

**Published:** 2020-07-22

**Authors:** Gaoming Li, Jingwen Zhang, Yaoting Hu, Yongning He

**Affiliations:** 1 School of Microelectronics, Faculty of Electronic and Information Engineering, Xi’an Jiaotong University, No. 28 Xianning West Road, Xi’an 710049, China; hyt961002@stu.xjtu.edu.cn; 2 School of Materials Science and Engineering, Xi’an Jiaotong University, No. 28 Xianning West Road, Xi’an 710049, China; 3 School of Electronic Science and Engineering, Xi’an Jiaotong University, No. 28 Xianning West Road, Xi’an 710049, China; jwzhang@mail.xjtu.edu.cn

**Keywords:** UV plasmonic enhancement, MgZnO, Al mesh

## Abstract

High-performance UV detectors are of great significance for various applications. Plasmonic structures enable great improvement of the performance of detectors. However, to push the plasmonic enhancement to photo response into the deep-UV region presents some challenges. In this work, we found that the optical properties of the supporting layer play important roles in achieving the optimal plasmonic enhancement. Therefore, we fully considered the dependence of the optical constants of the MgZnO supporting layer, which is a promising material to realize deep-UV photodetectors, on microstructure and crystalline quality, which are related to the fabrication method. Based on the optical constants, we designed an Al mesh plasmonic structure and fabricated it with a polystyrene monolayer as a mask. Finally, we demonstrated a three-times enhancement to photo response with UV radiation at 254 nm.

## 1. Introduction

High-performance UV detectors can be widely used in missile alarms, UV communication, ozone surveillance, fire alarms, and UV astronomy [1,2,3,4,5]. Therefore, designing and fabricating high-performance UV detectors that meet the various requirements from applications are of great importance. When the spectrum of detection goes into deep UV, such as the solar-blind range, the detectors could get a lower noise background and higher signal-to-noise ratio [6,7,8,9,10]. Usually, detecting spectrum is determined by the band gap of semiconductor material as the active layer. To push the detecting spectrum into deep UV needs a wider band gap for the semiconductor. MgZnO is a widely researched wide band gap semiconductor for UV detection and band gap engineering, since it has a continuously tunable and direct band gap, environmental friendliness, and feasibility [11,12,13,14]. However, like other wide band gap semiconductors, when the band gap turns very large, some problems such as phase segregation will emerge [11,15,16]. Semiconductors with a very large band gap always behave like insulators—when they are used to fabricate UV detectors, the performance needs to be improved.

In recent years, as the nanoscience and technology has rapidly developed, plasmonic structures can be employed to greatly enhance the performance of photoelectric devices [17,18,19,20,21]. Metallic nanoparticles, gratings, and other nanostructures with feature size in subwavelength have been reported to enable a strong enhancement to the photo response of photodetectors in IR, visible light, and the near-UV range of wavelength [22,23,24,25,26,27]. If the enhancement goes to even shorter wavelength, the feature size of the plasmonic structure will shrink further. And some challenges need to be well addressed. For example, it is very hard to accurately fabricate plasmonic structures with sizes of less than 100 nm. The fabrication methods, like electron beam lithography (EBL) and ion beam etching (IBE), are very expensive and not suitable for fabrication of a large area. Moreover, many materials have strong absorption in the deep-UV region, so the optical constants may change violently, and the variation of optical constants needs to be considered when one designs plasmonic structures.

In this paper, in order to push the plasmonic enhancement into the deep-UV region, we employed sputtered MgZnO film as the active layer. The dependence of optical constants on micro structure and crystalline quality is fully considered. In order to realize the optimal plasmonic enhancement, it is very necessary to take into account this dependence of optical properties. Based on the optical constants, we designed the Al mesh structures and simulated the optical properties of the structure of Al mesh on MgZnO film. The local surface plasmon resonance was verified. Then we used a polystyrene (PS) particle monolayer as a mask to fabricate the Al mesh. The width of the spacing area between two adjacent holes is 150 nm as we designed it. Compared to EBL, this method is cost-efficient and may enable large-area fabrication. Finally, we fabricated an Al mesh-enhanced photodetector and demonstrated an enhancement to photo current at a wavelength of 254 nm, which is in the deep-UV range. The enhancing factor can reach nearly three times. Our work paves the way to realize the plasmonic enhancement to photo response in the deep-UV region.

## 2. Materials and Methods

**Figure 1:**
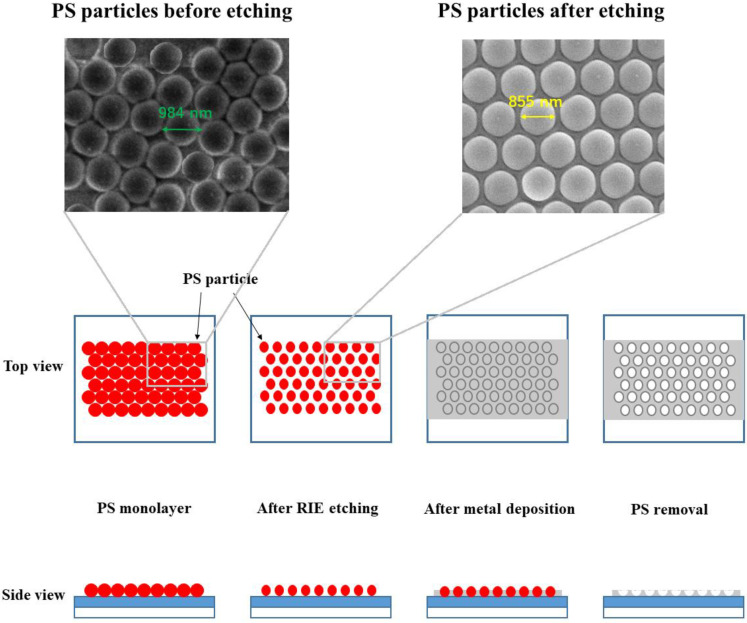
Fabrication process of Al mesh structure based on the polystyrene (PS) monolayer; the SEM image on the upper left shows the PS monolayer before reactive ion etching (RIE) and the upper right one shows the situation after etching. PS particles were on the MgZnO layer.

The plasmonic structure of Al mesh was fabricated with the help of a PS particles monolayer. We were inspired by Taguchi et al. to conceive this idea to fabricate the Al mesh with a feature size in subwavelength [28]. The fabrication process is shown in Figure 1. After the 70-nm-thick MgZnO film was sputtered on the quartz glass substrate, we did a PS particles monolayer deposition on the MgZnO film. Each particle is spherical and with the diameter of 1 micron. The deposition process was done in deionized (DI) water. We immersed the substrate in DI water. Then the PS suspension in ethanol with the concentration of 0.5% was slowly injected into the vessel where the water was contained. The injection rate is 3–4 μL/min. The PS particles were fully dispersed on the water surface and a monolayer was formed at the surface. Then the water drained out of the vessel very slowly through a pipe at the bottom, and the PS monolayer moved downwards as a whole. It took hours for the water-draining process. Once the water surface in the vessel was lower than the substrate, the PS monolayer would be transferred onto the substrate. From microscope and SEM images, we found that the monolayer was hexagonal close-packed. Then we employed oxygen plasma reactive ion etching (RIE) to etch the PS monolayer, and the PS particles would get smaller in size. The spacing between particles appeared and would increase with etching time. From the SEM image of PS particles after the etching shown in Figure 1, we can see that the diameter of the etched particle was about 850 nm. The original diameter of each PS particle before etching was about 1 µm. So, we deduced that the metallic spacing between the adjacent holes was about 150 nm. After etching, 500-nm-thick Al film was deposited on the PS monolayer by thermal evaporation. The last step to form Al mesh is to remove all the PS particles from the surface of MgZnO film by ultrasonic oscillation. This fabrication technique, in which the PS particles monolayer is used as a mask, is a promising method to realize continuous tuning of the width of mesh. On the basis of the Al mesh structure, we used photolithography and sputtering to fabricate the electrodes, thus the Al mesh-enhanced photodetector was finished. The characterization we did includes spectroscopic ellipsometry measurement for MgZnO film, optical measurement for the Al mesh structure with MgZnO film, and photoelectric property measurement for the Al mesh-enhanced photodetector.

## 3. Results and Discussion

### 3.1. Optical Constants of MgZnO

**Figure 2:**
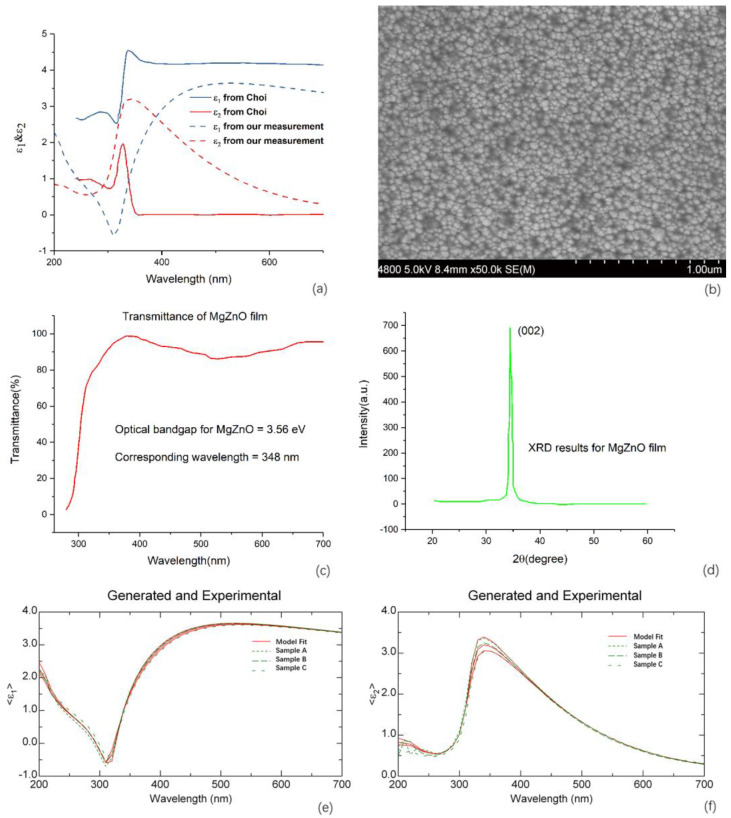
(**a**) Optical dielectric functions ε_1_ and ε_2_ of MgZnO films grown by two methods—one set of dashed lines are for the dielectric functions of our MgZnO film measured by ellipsometry, the other set of solid lines are for the dielectric functions of MgZnO film grown by spray pyrolysis from literature; (**b**) SEM image, (**c**) transmittance spectra, and (**d**) XRD measurement results of MgZnO film grown by sputtering in our experiment; (**e**) ε_1_ and (**f**) ε_2_ for our sputtering MgZnO films with different thicknesses, and samples A, B, and C are with thicknesses of 70, 120, and 160 nm, respectively.

Optical constants of the dielectric supporting layer are crucial parameters for plasmonic enhancement modulation and device design. Therefore, if we want to realize and optimize the plasmonic enhancement in a specific range of wavelength, we have to accurately acquire the optical constants of the supporting layer in such a range. Previous work has often assumed that the optical constants do not change with wavelength within the range in question, so the dispersion of optical constants was always neglected. In fact, within some wavelength ranges, especially where strong absorption happens, optical constants of the supporting layer are not invariants, and besides, they are dependent on the structural properties and crystalline quality. Ellipsometry is a common method to obtain the optical constants variation with wavelength. In Figure 2a, the real and imaginary parts of the dielectric function of MgZnO film is shown. In our spectral ellipsometry measurement, the incidence angle is 70 degrees, and the measuring spectral range is from 200 to 700 nm. After we acquired the amplitude component and phase difference for the complex reflection ration, we did the fitting using the Lorentz oscillator model. The mean square error is rather low so that it indicates that the results we obtained are reliable. As Figure 2a shows, the results of the optical constants of MgZnO film derived from our spectroscopic ellipsometry measurement present apparent discrepancies with the published results from Choi et al. [29]. In their paper, the MgZnO film was grown by ultrasonic spray pyrolysis, which is different from our fabrication method, namely sputtering. This is supporting evidence for the conclusion that the optical constants of MgZnO are highly dependent on the microstructure and crystalline quality, hence the fabrication method.

The transmittance spectra are shown in Figure 2c. The band edge absorption can be used to estimate the optical band gap of the measured material. Based on the linear approximation for the relationship between Mg content in MgZnO and band gap, we can deduce that the atomic percentage of Mg content is 7.6% for our sample. The MgZnO target we used for sputtering is with the Mg content of 10%. It makes sense that Mg concentration of the MgZnO sample fabricated by sputtering is lower than that of the target. The sputtered MgZnO film is polycrystalline and we can recognize grains and boundaries in the SEM image shown in Figure 2b. The grain size is in the scale of about 30 nm. The X-ray diffraction (XRD) measurement result of our MgZnO film is shown in Figure 2d. The sharp spike corresponding to the (200) lattice plane indicates that the crystalline quality of our sample is good. No doubt, there exist differences in grain size, crystallinity, and surface morphology of the MgZnO films grown by sputtering and spray pyrolysis. And these differences are the reasons why ε_1_ and ε_2_ of MgZnO films grown by two methods exhibit a large discrepancy. In order to show that the same fabrication method brings similar optical properties, we measured ε_1_ and ε_2_ for our sputtered MgZnO films with different thicknesses. In Figure 2e,f, the thicknesses for samples A, B, and C are 70, 120, 160 nm, respectively. Their similar optical properties just verified our conclusion.

### 3.2. Design of Al Plasmonic Structure and Simulation

**Figure 3:**
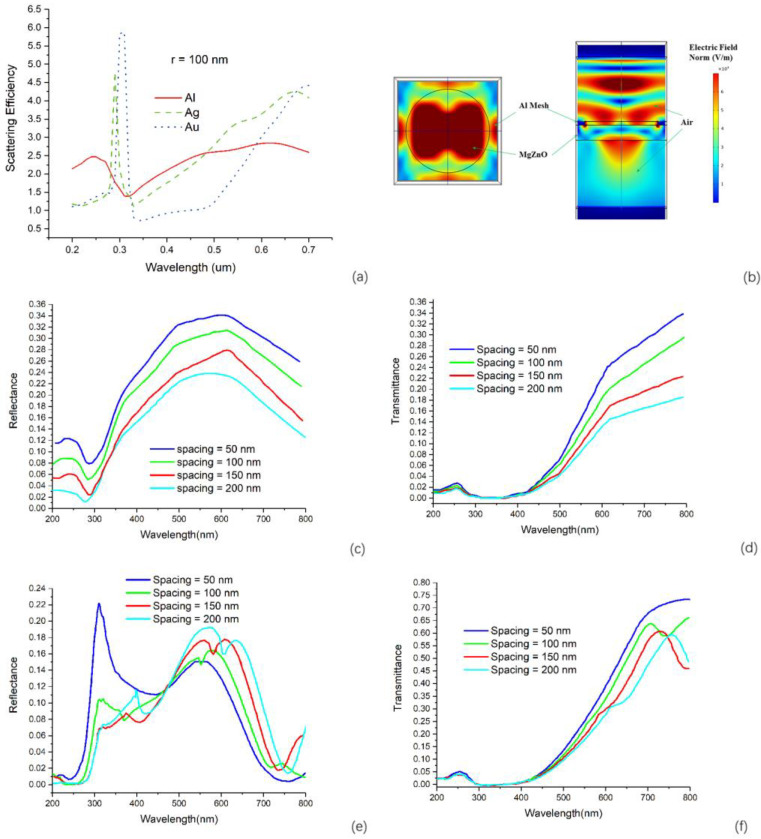
(**a**) Calculated scattering efficiency of Au, Ag, Al particles with radius of 100 nm in MgZnO; (**b**) electric field distribution in Al mesh structure within one hole, which is a unit cell for our simulation, the left one is the top view and right one is the cross-section view, the width of spacing is 150 nm; (**c**) simulated reflectance and (**d**) transmittance spectra of the structure of Al mesh on MgZnO film, the simulation is based on ε_1_ and ε_2_ from our ellipsometry measurement results, the spacing width was swept from 50 to 200 nm; simulated (**e**) reflectance and (**f**) transmittance spectra of the structure of Al mesh on MgZnO film using optical permittivities from reference [29].

**Figure 4:**
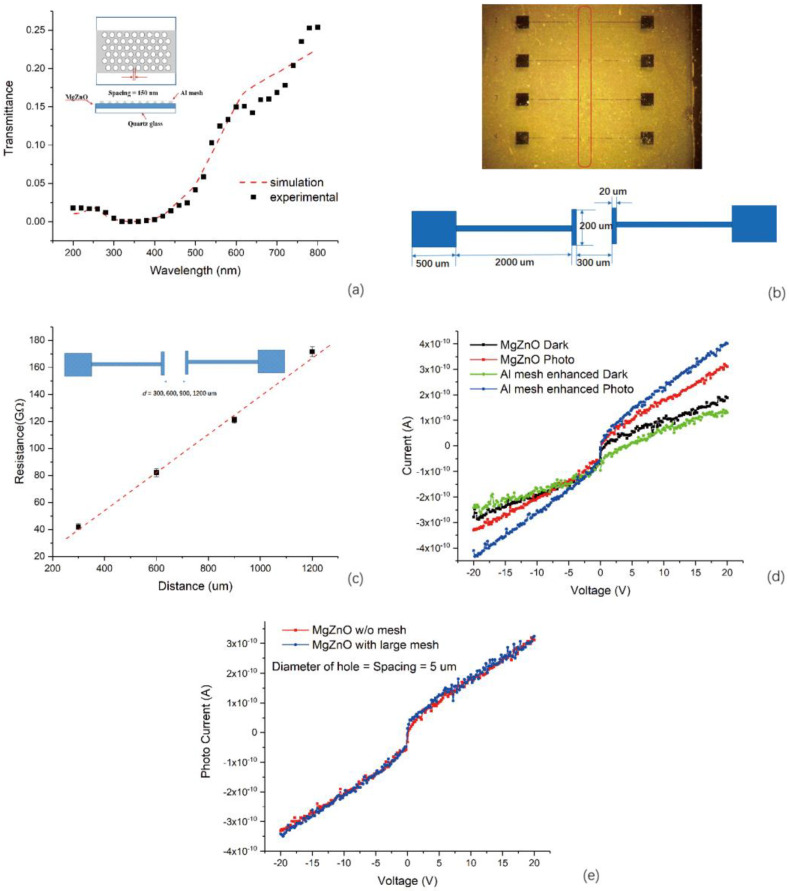
(**a**) Experimental data vs. simulated results for transmittance for the structure of Al mesh on MgZnO—the size and structural schematic are shown in the inset; (**b**) the upper figure is the microscope image of the Al mesh-enhanced detector, Al mesh is in the area marked by the red rectangle, the lower figure shows the size information of electrodes; (**c**) measured resistance when the distance between electrodes varies from 300 to 1200 µm with a step of 300 µm, the resistance is proportional to the distance, the inset shows how to define the distance; (**d**) I–V curves in dark and under UV illumination for the MgZnO control detector and Al mesh-enhanced MgZnO-based detector; (**e**) photo current for MgZnO control detector and normalized photo current for the MgZnO-based detector with large mesh, the spacing is equal to the diameter of the hole which is 5 µm.

MgZnO film plays two roles in our photodetectors. One is as the supporting layer that is contiguous with metallic plasmonic structure. The other is as an active layer where photo carriers are generated. After we investigated the optical properties of MgZnO film, we were able to design the metallic plasmonic structures to achieve an optimal enhancement of the performance of the plasmonic enhanced MgZnO-based photodetector, which also means to maximize its function as the first role. We simulated the scattering efficiency of a metallic spherical particle with a radius of 100 nm in the supporting layer of MgZnO. The metallic materials we used are Al, Ag, and Au. Although nanoporous metallic materials also showed some interesting results in UV plasmonics [30,31,32], we didn’t consider the porosity here. The optical constants of Al, Ag, and Au, are from literature [33,34]. From the results shown in Figure 3a, we see that the peak wavelength of scattering efficiency takes the order that Al < Ag < Au. It coincided with other published work, which indicated that Al is a good choice for UV plasmonics [35,36]. As we all know, researchers have demonstrated extraordinary transmission and light beaming through metallic mesh or hole arrays due to plasmonic resonance. Actually, these metallic meshes also could be employed to realize an enhancement to the photodetector if the size of some parts of the mesh structure is in subwavelength range. Just as the inset of Figure 4a shows, the width of spacing between two adjacent holes can be adjusted to subwavelength range. We did a simulation about the Al mesh structure on the MgZnO film to see how the width of spacing influences the plasmonic resonance and the optical properties like transmittance and reflectance. We chose one hole as a unit cell, which can be found in Figure 3b. The boundary conditions were set using Floquet periodicity boundary conditions. The excitation port and listener port were placed more than two wavelengths away from the Al mesh and MgZnO film. We did a parametric sweep by changing the width of spacing from 50 to 200 nm with a step of 50 nm. We calculated the transmittance and reflectance spectra of the structure, which consists of Al mesh and MgZnO film. In the simulation, the dependence of optical properties on wavelength for both Al and MgZnO is fully considered. We used the ellipsometry measurement results for this optically flat MgZnO in the simulation, and the optical properties of Al are from literature [34]. As we can see from Figure 3c,d, the plasmonic resonance is more apparent in the reflectance spectra compared to transmittance spectra. The dip around 280 nm in the reflectance spectra is due to enhanced scattering and absorption resulted from plasmonic resonance. The dip blue-shifts when the spacing reduces, this result matches our expectation based on the relationship between the size of plasmonic structure and wavelength of plasmonic resonance. However, this shift of wavelength of plasmonic resonance is not so large in our simulation. Therefore, if we want to realize the plasmonic enhancement in the UV range, especially below the wavelength of 280 nm, based on our simulation results the choice for the width of spacing should be less than 200 nm. Based on the electric field distribution of our simulation results in Figure 3b, the local field enhancement under the spacing area also confirms the local surface plasmon resonance. Figure 3e,f shows the simulated reflectance and transmittance of Al mesh on MgZnO film using the permittivities from reference [29]; other parameters used in the simulation are the same as those used in Figure 3c,d. Comparing these two sets of simulation results, we can find that the reflection is more sensitive to the optical permittivities. Based on the previous discussion, we can draw the conclusion that different estimation of optical permittivities of MgZnO would entail different structural optimizations, and it is very important to take into account the optical properties of the supporting layer for achieving the optimal plasmonic enhancement.

### 3.3. UV Plasmonic Enhancement to Photo Response

The Al mesh structure was fabricated by a method in which a PS particle monolayer was used as a mask—the details of the fabrication process is discussed in the experimental part. From the SEM image of Al mesh shown in Figure 1, we can see that the diameter of the hole was about 850 nm. The original diameter of each PS particle before etching was 1 µm. So, we deduced that the metallic spacing between the adjacent holes was about 150 nm. After the fabrication of Al mesh, we measured the transmittance spectra of Al mesh on MgZnO film. As Figure 4a shows, the experimental data agreed well with the simulation results. Then we used photolithography and sputtering to fabricate the Cr electrodes to form an Al mesh-enhanced photodetector. The optical microscope image of the detector and the size of the structure is shown in Figure 4b. Each pair of electrodes consisted of two big squares at the end, which were used as bonding pads. The Al mesh was in the area between two parallel bars which were marked out by the red rectangle. The photodetector with same electrodes, however without Al mesh between electrodes, was set as a control. We measured the dark currents for both photodetectors. As the results in Figure 4d show, the dark current for the Al mesh-enhanced photodetectors was smaller compared to the MgZnO control detector. The reason for the dark current decrease is that a depletion layer at the surface of MgZnO is formed where the Al mesh contacts the MgZnO film. As we all know, when metal contacts a semiconductor, the charge carriers will flow, due to the difference of Fermi level between the metal and semiconductor. Then a potential barrier at the interface and a depletion layer at the surface of the semiconductor is formed. The depletion layer results in the increase of sheet resistance of the surface of the MgZnO film. We measured the resistance when the distance between the parallel bars varies. And Al mesh structure covered the area between the parallel bars. As Figure 4c shows, that the resistance is linearly related to the distance is good proof to support our claim.

We used a mercury lamp with wavelength of 254 nm as the light source to measure the photo response. The photo current for the Al mesh-enhanced photodetector and the MgZnO control detector is shown in Figure 4d. Compared with dark current, the photo current increased for both detectors. However, the photo current for Al mesh-enhanced photodetector is apparently greater than the control one. Therefore, we assume that we realized the localized surface plasmon (LSP) enhancement to photo current through the Al mesh structures at the wavelength of 254 nm. As we all know, the plasmon’s effect is highly sensitive to the size, which should be in the range of subwavelength. Based on this knowledge we designed another experiment to exclude other effects that may cause an increase of photo current. We used photolithography patterned with a similar mesh structure but with a much larger size. The diameter of the hole is equal to the spacing which is 5 µm. Apparently, Al mesh structure in this size cannot generate the plasmonic effect in the UV region. Then we measured the photo current for this MgZnO-based detector with large mesh. Because the illumination area reduced a lot when the large Al mesh covered the surface MgZnO layer, we normalized the photo current to the sensing area for this detector. Compared with the normal detector, we observed no increase for the photo current as Figure 4e shows. This result indicates that the photo current increase is mainly due to the localized surface plasmon effect. Otherwise, we should have witnessed an increased photo current for the detector with large Al mesh. Moreover, the simulation results about the localized field enhancement shown in the previous part also support our conclusion. We used the following equation to calculate enhancement factor,

(1) $$E=\frac{I_{ph}\left(U\right)-I_{d}\left(U\right)}{I_{ph0}\left(U\right)-I_{d0}\left(U\right)}$$

where *I_ph_*(*U*), *I_d_*(*U*) is photo current and dark current for Al mesh enhanced photo detector at the bias of *U*, respectively. and *I_ph_*_0_(*U*), *I_d_*_0_(*U*) is photo current and dark current for the control detector at the bias of *U*, respectively. When we use the value at *U* = 10 V, the enhancement factor is equal to 2.98. Though the plasmonic resonance wavelength is about 280 nm according to the reflection spectra, we still demonstrated a photo current enhancement due to LSP at 254 nm. The possible reason may be that the enhancement peak is wide in the spectrum.

## 4. Discussion

In this paper, we have fabricated the MgZnO film by sputtering. Based on the spectroscopic ellipsometry measurement results, we find that the optical constants of the MgZnO film are dependent on the microstructure and crystalline quality, which are related to fabrication method. If we want to achieve the optimal enhancement of the plasmonic enhanced photodetector, considering the variation of optical properties of supporting layers is quite important. Then we designed a plasmonic structure of Al mesh on the MgZnO film and made a simulation for its optical properties using the optical constants we derived. The simulation results show that when the metal spacing between adjacent holes is in the subwavelength range, the strong local surface plasmon resonance will be generated under the metal spacing area and the plamonic resonance wavelength is about 280 nm. We finally chose 150 nm for the width of spacing. Then a fabrication technique in which a PS particle monolayer was used as a mask was employed to fabricate the Al mesh. From the SEM image, we see that the width of spacing is about 150 nm as we designed, and the holes are in hexagonal closed-pack alignment. The measured transmittance of the structure of Al mesh on MgZnO film agrees well with the simulation results. It verifies the validity of our design and simulation. Then we used photolithography and sputtering to fabricate the electrodes to form Al mesh-enhanced photodetectors. Compared with the MgZnO control detector, the dark current for Al mesh-enhanced detector decreased due to the occurrence of a depletion layer. The depletion layer is at the surface of MgZnO where the Al mesh covered it. That the resistance between electrodes is linearly related to the distance between them supports our claim. Under UV illumination at 254 nm, the photo current for Al mesh-enhanced photo detector increased due to LSP resonance. The enhancement factor could reach nearly three times. Thus, we demonstrated an LSP enhancement to photo current at 254 nm, which is in the deep-UV region. Speaking of UV plasmonic enhancement for the MgZnO-based photon detector, Guo et al. realized the responsivity enhancement at a wavelength of 325 nm using Au nanoparticles, the peak enhancement being about 2.6 times at 30 V [37]. Lee et al. demonstrated a 1.365-time detectivity enhancement within the wavelength range from 300 to 330 nm by exploiting Ni/Au nanomesh [38]. Pt nanoparticles were also employed to realize two-time enhancement at about 310 nm [39]. Therefore, compared with these published works, our results showed the advantages both in the enhancing wavelength and the enhancement factor. Our result is of significance to push the plasmonic enhancement to photo detectors into the deep-UV region.
